# Quantitative phosphoproteomics uncovers synergy between DNA-PK and FLT3 inhibitors in acute myeloid leukaemia

**DOI:** 10.1038/s41375-020-01050-y

**Published:** 2020-10-16

**Authors:** Heather C. Murray, Anoop K. Enjeti, Richard G. S. Kahl, Hayley M. Flanagan, Jonathan Sillar, David A. Skerrett-Byrne, Juhura G. Al Mazi, Gough G. Au, Charles E. de Bock, Kathryn Evans, Nathan D. Smith, Amanda Anderson, Brett Nixon, Richard B. Lock, Martin R. Larsen, Nicole M. Verrills, Matthew D. Dun

**Affiliations:** 1 Cancer Research Program, Hunter Medical Research Institute, School of Biomedical Sciences and Pharmacy Faculty of Health and Medicine, University of Newcastle, Callaghan, NSW 2308 Australia; 2 Calvary Mater Newcastle Hospital, Waratah, NSW 2298 Australia; 3 NSW Health Pathology, John Hunter Hospital, Lookout Road, New Lambton Heights, NSW Australia; 4 School of Medicine and Public Health Faculty of Health and Medicine, University of Newcastle, Callaghan, NSW 2308 Australia; 5 Viruses, Infections/Immunity, Vaccines and Asthma Program, Hunter Medical Research Institute, and School of Biomedical Sciences and Pharmacy, Faculty of Health and Medicine, University of Newcastle, Callaghan, NSW 2308 Australia; 6 Priority Research Centre for Reproductive Science, School of Environmental and Life Sciences, University of Newcastle, Callaghan, NSW 2308 Australia; 7 Children’s Cancer Institute Australia, School of Women’s and Children’s Health, University of New South Wales, Sydney, NSW Australia; 8 Analytical and Biomolecular Research Facility, Advanced Mass Spectrometry Unit, University of Newcastle, Callaghan, NSW 2308 Australia; 9 Department of Molecular Biology and Biochemistry, Protein Research Group, University of Southern Denmark, Odense, 5230 Denmark

**Keywords:** Acute myeloid leukaemia, Preclinical research

## To the Editor:

Acute myeloid leukaemia (AML) is the most common and aggressive form of acute leukaemia in adults. The most common driver mutations in AML are activating mutations in the FMS-like tyrosine kinase 3 (FLT3) gene, occurring in approximately one third of cases [1]. Internal tandem duplication (FLT3-ITD) mutations in the FLT3 juxtamembrane domain are the most common (~25%), and are associated with genomic instability, and intermediate-adverse prognosis [2]. Point mutations in the FLT3 activation loop, most commonly at amino acid D835, occur in ~8% of AML patients and their prognostic effect remains to be defined.

**Fig. 1:**
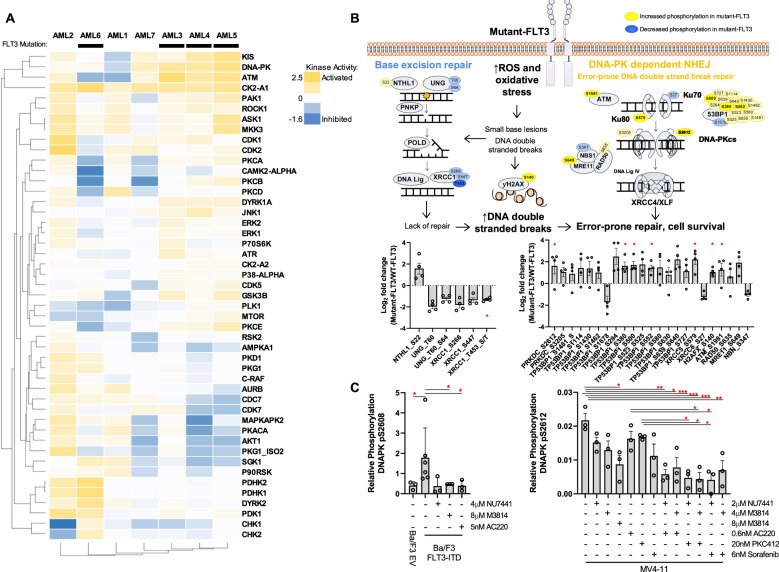
Quantitative phosphoproteomic profiling of human AML blasts identifies phosphorylation of DNA repair, and growth and survival signalling pathways. The phosphoproteome of seven human AML blast samples was quantified by iTRAQ mass spectrometry. **a** Kinase activity profile of AML blast samples was determined by individual kinase substrate enrichment analysis (KSEA), performed on each sample separately using the mass spectrometry median-normalised data. Colour scale indicates PHOXTRACK kinase enrichment score, with a positive value predictive of kinase activation and a negative value predictive of inhibited kinase activity. **b** DNA repair pathways displayed altered phosphorylation in mutant-FLT3 AML blasts, with increased phosphorylation of proteins (yellow) within the Non Homologous End Joining pathway and decreased phosphorylation (blue) within the Base Excision Repair pathway analysed using 2-way ANOVA *p* = 0.0397 and *p* = 0.0436, respectively. Phosphosites with a greater than 2-fold change in abundance are shown. Darker shading indicates individual phosphorylation sites that are statistically significantly different between wildtype and mutant-FLT3 AML samples, as analysed using one-tailed test *p < 0.05. **c** DNA-PKcs phosphorylation levels were assessed by targeted mass spectrometry in cell lines treated for 1 h with DNA-PK inhibitors (NU7441, M3814), FLT3 inhibitors (sorafenib, midostaurin, AC220), or their combination, as indicated. *p < 0.05, **p < 0.01, *n* = 3.

The landscape of treatment options for AML is rapidly changing, however there remains limited durable treatment options for molecular subtypes such as mutant-FLT3 AML. To identify novel therapeutic targets, we undertook quantitative phosphoproteomic profiling of primary AML blasts (Supplementary Methods) [3]. Across seven patient samples (3 × wildtype-FLT3, 4 × mutant-FLT3; Tables S1 and S2), 4345 unique phosphosites were identified from 1994 proteins, with expected ratios of serine, threonine, and tyrosine sites [pS:pT:pY 88.9%:10.6%:0.6%]. The top pathways phosphorylated in AML blasts were growth and survival signalling pathways (ERK/MAPK, AMPK signalling, Fig. S1) and DNA damage repair signalling pathways (ATM signalling, DNA double strand break (DSB) repair, Fig. S1). Kinase substrate enrichment analysis (KSEA) revealed activation of the cell cycle and apoptosis regulator, Casein Kinase 2 (CK2-A1), in all blast samples (Figs. 1a and S2). Increased CK2-A1 expression has been demonstrated in a range of haematological cancers, including AML; driving cell proliferation, survival, and drug resistance [4]. Further validating our approach, Glycogen Synthase Kinase 3β (GSK3β), a downstream regulator of mutant-FLT3 proliferative signalling [5], and Cyclin-Dependent Kinase 5 (CDK5), an AML drug target [6], showed activation in mutant-FLT3 samples (Fig. 1a). The serine/threonine protein kinase KIS, a regulator of proliferation in leukaemia cells [7]; and the DSB repair protein kinases DNA-dependent Protein Kinase (DNA-PK) and Ataxia Telangiectasia-Mutated (ATM), were also activated in the majority of samples (Figs. 1a and S2). In mutant-FLT3 samples compared to wildtype-FLT3, 143 peptides displayed a significant twofold increase and 90 peptides displayed a significant twofold decrease (Fig. S3). Pathway enrichment analysis revealed increased phosphorylation of proteins involved in the error-prone DNA-PK-dependent Non Homologous End Joining (NHEJ) pathway in mutant-FLT3 AML patient samples, compared to wildtype-FLT3 patients (Figs. 1b, S1B, and S4). This included core NHEJ proteins DNA-PKcs (PRKDC), XRCC5, XRCC4, and 53BP1 (Figs. 1b and S4), suggesting NHEJ pathway activation in mutant-FLT3 samples. In support of this, mutant-FLT3 patient samples displayed increased phosphorylation of the DNA-PK activating autophosphorylation site, S2612(*p* = 0.047, Fig. 1b), analogous to previous results in *FLT3-*, *NRAS-*, and *BRAF*-mutant AML [8]. In addition, phosphorylation of Base Excision Repair (BER) pathway proteins were decreased in mutant-FLT3 compared to wildtype-FLT3 AML patients (Figs. 1b and S4), which has not been previously reported.

To investigate if DNA-PKcs phosphorylation is linked to FLT3 activity, we used an established Ba/F3 mouse cell line model of FLT3 signalling [9]. Phosphorylation of DNA-PKcs at S2608 (homologous to the human autophosphorylation site S2612) was significantly higher in Ba/F3 cells transduced with FLT3-ITD, compared to control empty vector cells (EV, *p* = 0.048, Fig. 1c). As expected, S2608-DNA-PK phosphorylation in Ba/F3-FLT3-ITD cells was significantly decreased with DNA-PK inhibitor treatment (M3814 *p* = 0.024; Fig. 1c). Importantly, S2608-DNA-PK phosphorylation was significantly decreased by treatment with the FLT3 inhibitor, AC220 (*p* = 0.024, Fig. 1c); suggesting that DNA-PK activity is linked with FLT3 activity. Similarly, S2612-DNA-PK phosphorylation in human FLT3-ITD AML MV4-11 cells was decreased following DNA-PK inhibition (M3814 *p* = 0.017) and further decreased following combined FLT3 and DNA-PK inhibition (NU7441 + AC220 *p* = 0.003, M3814 + AC220 *p* = 0.018, NU7441 + midostaurin *p* = 0.003, M3814 + midostaurin *p* = 0.004, NU7441 + sorafenib *p* = 0.004, M3814 + sorafenib *p* = 0.01, Fig. 1c).

To investigate the therapeutic potential of inhibiting DNA-PK in AML, we assessed cell survival using the DNA-PK/mTOR inhibitor, NU7441 (DNA-PK_IC50_ = 0.014 μM, mTOR_IC50_ = 1.7 μM) and the potent, selective DNA-PK inhibitor M3814 (DNA-PK_IC50_ = 0.0006 μM). M3814 is currently in clinical trial for advanced solid tumours (NCT02516813), and in combination with chemotherapy for relapsed/refractory AML (NCT03983824). Isogenic Ba/F3 cell lines dependent on FLT3 signalling for growth (FLT3-D835V, -D835Y, -ITD, and -WT cells grown in FLT3 ligand (FL)) were more sensitive to NU7441 compared to either EV cells or WT-FLT3 cells grown in IL-3 (Table S3). Similarly, Ba/F3 lines dependent on FLT3 signalling were sensitive to M3814, while EV cells and WT-FLT3 cells grown in IL-3 did not reach IC50 in the dose range tested (Table S3). Human AML cell lines with either *FLT3* (MV4-11, MOLM13) or *NRAS* (HL60, THP1) mutations all displayed dose-dependent sensitivity to NU7441 and M3814 (Fig. S5).

**Fig. 2:**
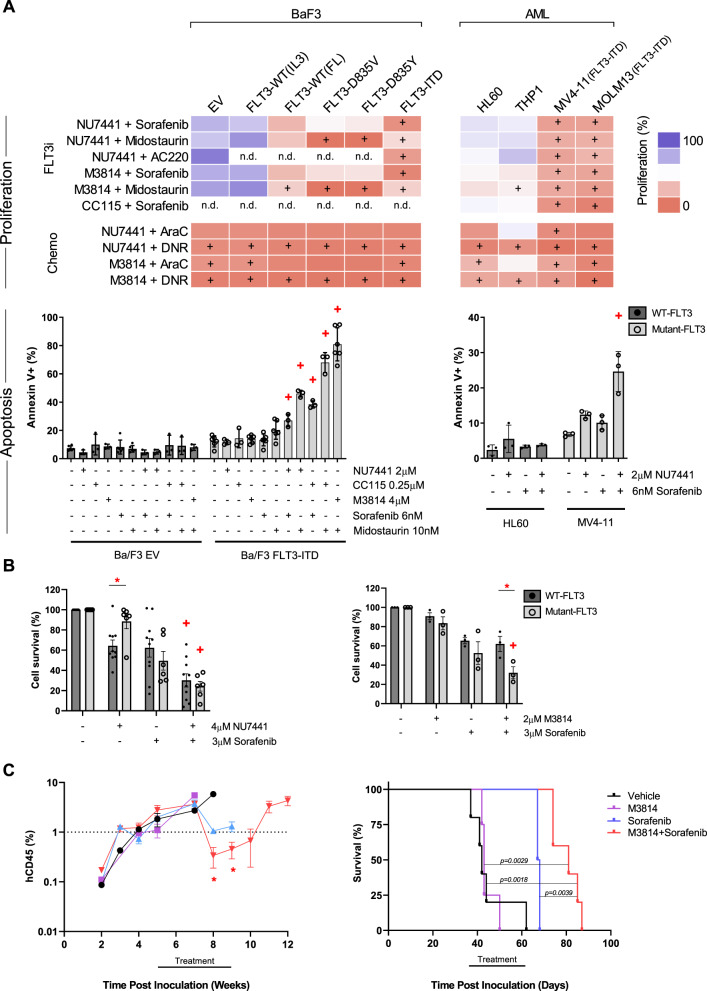
DNA-PK and FLT3 inhibitors are synergistic and potent in mutant-FLT3 AML. **a** Proliferation and apoptosis assessed in Ba/F3 and AML cell lines. *Upper*: Cell survival assessed by resazurin assay, after 72 h treatment with DNA-PK inhibitors (2 µM NU7441, 4 µM M3814, 0.5 µM CC115) and FLT3 inhibitors (0.6 nM AC220, 6 nM sorafenib, 20 nM midostaurin (AML lines), 5 nM midostaurin (Ba/F3 lines)). +, Synergistic drug combinations, determined by the method of Chou Talalay (Table S4). nd, not determined. *Lower*: Apoptosis was assessed by Annexin V^+^ flow cytometry, after 48 h treatment with the indicated inhibitors. +, Synergistic drug interaction, calculated using the fractional product method of Webb. *n* = 3 + SEM. **b** Viability of wildtype-FLT3 and mutant-FLT3 AML patient blast samples after 24 h treatment with the indicated DNA-PK (NU7441, M3814) and FLT3 (sorafenib) inhibitors (Table S5). Cell survival was determined by Annexin V and PI negativity. *, *p* < 0.05 between wildtype-FLT3 and mutant-FLT3 groups. +, Synergistic drug interaction, calculated using the fractional product method of Webb using the group mean. *n* = 3 + SEM. **c** NRG mice were transplanted with FLT3-ITD MV4-11 cells. Once engraftment reached 2% in the peripheral blood, mice were randomised and treated 5 days/week for 4 weeks. Treatment with vehicle (*n* = 5), 150 mg/kg M3814 (*n* = 3), 5 mg/kg sorafenib (*n* = 4), or 150 mg/kg M3814 + 5 mg/kg sorafenib (*n* = 5) commenced 5 weeks post inoculation of MV4-11 cells. *Left*, Leukaemia burden in the peripheral blood was measured by flow cytometric analysis of the levels of human CD45 (hCD45) positive cells as a percentage of total human and mouse CD45+ cells. *p < 0.05. *Right*, Kaplan Meier survival analysis revealed a significant survival advantage in mice treated with M3814 combined with sorafenib. Log-rank test: vehicle vs M3814 + sorafenib, *p* = 0.0018; M3814 vs M3814 + sorafenib, *p* = 0.0029, sorafenib vs M3814 + sorafenib, *p* = 0.0039.

We next tested a panel of FLT3 inhibitors in combination with DNA-PK inhibitors. Mutant-FLT3 cell lines were sensitive to the selective type II FLT3 inhibitor AC220, combined with NU7441, which effected a synergistic reduction in cell growth in the mutant-FLT3 cell lines (MV4-11, MOLM13, and Ba/F3 FLT3-ITD) but not the WT-FLT3 lines (THP1, HL60, BaF3-EV) (Figs. 2a and S5, and Table S4). Similarly, DNA-PK inhibitor combinations with the type I FLT3 inhibitor midostaurin (PKC412), were potent and synergistic in mutant-FLT3 cells (Figs. 2a and S5, and Table S4). Interestingly, DNA-PK inhibitor combinations with the type II FLT3 inhibitor sorafenib, were more potent than combinations with midostaurin in FLT3-ITD cells, possibly due to dual FLT3 and MAPK inhibition (Figs. 2a and S5, and Table S4). As expected, FLT3-D835 mutant lines were resistant to the type II inhibitor sorafenib, and no synergy was observed (Fig. 2a and Table S4), suggesting that synergy is dependent on FLT3 inhibition. In comparison, DNA-PK inhibitor combinations with DNA-damaging AML chemotherapeutics, cytarabine and daunorubicin, inhibited proliferation in all lines, irrespective of FLT3 status (Figs. 2a and S6, and Table S4).

The combination of NU7441 and sorafenib enhanced the accumulation of cells in G1 phase and significantly reduced the proportion of cells in S and G2M phases, in FLT3-ITD MV4-11 cells but not WT-FLT3 HL-60 cells (Fig. S7). NU7441 and sorafenib concurrently increased the proportion of MV4-11 cells in sub-G1, indicating cell death (Fig. S7). This was confirmed by Annexin V analysis, which revealed a synergistic induction of apoptosis with NU7441 combined with sorafenib, in MV4-11 but not HL60 cells (Fig. 2a). Similarly, DNA-PK inhibitors (NU7441, CC-115, or M3814) and FLT3 inhibitors (sorafenib, midostaurin) synergistically induced apoptosis in Ba/F3 FLT3-ITD cells, but not Ba/F3 EV cells (Fig. 2a).

To investigate the clinical relevance of DNA-PK and FLT3 inhibitor combinations, drug sensitivity was assessed by Annexin V flow cytometry in human AML blasts ex vivo. NU7441 combined with sorafenib was synergistic in both wildtype and mutant-FLT3 blasts (Figs. 2b and S8A). In contrast, mutant-FLT3 blasts were more sensitive than wildtype-FLT3 blasts to the more potent DNA-PK inhibitor M3814 combined with sorafenib, with the combination effecting synergy (*p* = 0.041, Figs. 2b and S8B).

The potentiation of DNA-PK and FLT3 inhibitors in mutant-FLT3 cells was further shown in an *in vivo* preclinical AML model. FLT3-ITD MV4-11 cells, stably expressing luciferase, were engrafted in wildtype-DNA-PK Nod-Rag-Gamma (NRG) mice (Supplementary Methods). Once mean leukaemia burden reached 2% in the peripheral blood, mice were randomised to receive vehicle, M3814 (150 mg/kg), sorafenib (5 mg/kg), or M3814 combined with sorafenib (150 mg/kg M3814 + 5 mg/kg sorafenib). As a monotherapy, M3814 had no effect on leukaemia burden (Fig. 2c). However, sorafenib monotherapy and M3814 combined with sorafenib reduced the proportion of leukaemia cells in the peripheral blood, with a deeper and more sustained response achieved with the combination (Fig. 2c). Bioluminescence measurements also demonstrated a deeper remission of systemic leukaemia burden in the combination group (Fig. S9). Consequently, M3814 combined with sorafenib led to a significant survival benefit; with a median survival of 81 days, compared to 67.5 for mice receiving sorafenib alone (*p* = 0.0039), 43 days for mice receiving M3814 alone (*p* = 0.0029), and 42 days for mice receiving vehicle (*p* = 0.0018, Fig. 2c).

Herein, the increased phosphorylation of DNA-PK-dependent NHEJ proteins, and decreased phosphorylation of BER pathway proteins, identified by phosphoproteomics in mutant-FLT3 AML blasts (Fig. 1b) together uncovers a potential mechanistic explanation for the mutation signatures reported in mutant-FLT3 AML. FLT3-ITD AML displays a C > A and T > G transversion signature [10] consistent with a lack of repair of oxidative DNA damage lesions, which are substrates for BER. Mutant-FLT3 cells have increased error-prone DNA DSBR [11, 12], which has not been previously linked with activation of DNA-PK-dependent NHEJ. Mutant-FLT3 AML patients display a high rate of cytogenetic evolution [13], and a high frequency of rare structural chromosomal variations at relapse [14], consistent with the mutation signature of over-active DNA-PK-dependent NHEJ [15].

Collectively, we have shown the utility of quantitative phosphoproteomic profiling of AML blasts for identifying activated pathways and guiding the rational selection of drug target combinations. As DNA-PK displayed activation in the majority of AML blast samples, the use of multikinase inhibitors in combination with DNA-PK inhibitors may provide therapeutic benefit in a range of AML subtypes, not limited to mutant-FLT3 AML. Although the mechanism linking FLT3 with DNA-PK activation remains to be determined, our studies demonstrate that DNA-PK is an attractive therapeutic target in AML. Our preclinical results support the clinical evaluation of DNA-PK inhibitors in combination with FLT3 inhibitors as a novel therapeutic strategy for mutant-FLT3 AML.

## Supplementary information

Supplementary Methods

Supplementary Figures

Supplementary Data
