# Supplementary material for: Quantitative phosphoproteomics uncovers synergy between DNA-PK and FLT3 inhibitors in acute myeloid leukaemia
Source: Leukemia. 2020 Oct 16;35(6):1782–7. doi: 10.1038/s41375-020-01050-y (PMC8179851; doi:10.1038/s41375-020-01050-y)
Supplement: Supplementary file 1 — Supplementary Methods [file 41375_2020_1050_MOESM1_ESM.docx]

**Supplementary materials and methods**

**Patient samples**

Treatment naïve *de novo* AML patients at diagnosis (Tables S1, Figure 2) were recruited through the Calvary Mater Newcastle, and the Royal Adelaide Hospital, in accordance with institutional guidelines. Studies were approved by the human ethics committees of the Hunter New England Area Health service, the University of Newcastle, and the Royal Adelaide Hospital. Written informed consent was obtained from all participants. Karyotype and FLT3 status was determined through routine pathology tests. Leukaemic blasts were isolated from bone marrow or peripheral blood samples using Lymphoprep density gradient medium (StemCell; Vancouver, Canada) and SepMate tubes (StemCell), as previously described (1). Annexin V assays described below were performed upon sample collection, prior to receipt of gene sequencing results; therefore these experiments were performed blinded to the mutations present in each sample (Figures 2B, Table S5).

**Next generation sequencing (NGS)**

DNA was extracted from AML patient blast samples using either the RNA/DNA/protein purification plus kit (Norgen Biotek; Thorold, ON, Canada) or the Wizard SV Genomic DNA Purification System (Promega), as per manufacturer’s instructions. Purified DNA was quantified using a Qubit fluorometer (Thermo Fisher; Carlsbad, CA, USA). DNA samples were sequenced using the Myeloid Solution 30-gene panel (Sophia Genetics; Boston, MA, USA) and an Illumina Mi-Seq (San Diego, CA, USA), as per manufacturer’s instructions. Data files were analysed using Sophia DDM and Alissa clinical informatics (Agilent; Santa Clara, CA, USA) platforms. Mutations identified in each patient are listed in Table S1.

**Cell lines**

Four human AML cell lines were used in this study; two FLT3-ITD (MV4-11^ITD/ITD^, MOLM13^WT/ITD^), and two wildtype (WT) FLT3, mutant-NRAS (HL60, THP1). HL60 and MOLM13 were kind gifts from Leonie Ashman (University of Newcastle), and Jason Powell (University of South Australia), respectively. MV4-11 and THP1 were a kind gift from Dr Kyu Tae Kim (University of Newcastle), and MV4-11 cells transduced with firefly luciferase (MV4-11-luc) were a kind gift from Prof Jan Cools (KU Leuven, Belgium). Cells were maintained in standard culture conditions (5% CO2, 37^o^C) in 10% FBS, 20mM HEPES (N-2-hydroxyethylpiperazine-N'-2-ethanesulfonic acid), and 2mM L-Glutamine, in either DMEM (MV4-11, HL60) or RPMI (MOLM13, THP1). β-Mercaptoethanol was added to THP1 culture medium at a final concentration of 0.05mM.

Murine haematopoietic progenitor Ba/F3 cells transduced with human wildtype (WT) FLT3, FLT3-ITD, FLT3-D835Y, FLT3-D835V, or an empty vector (EV), were previously generated in our laboratory (1). All Ba/F3 lines were maintained in RPMI medium as above, with the addition of 4ng/mL murine IL-3 (EV and FLT3-WT lines; Biolegend, CA, USA), or 50ng/mL human FLT3-ligand (FLT3-WT cells; Biolegend). Ba/F3 FLT3-mutant lines are factor-independent and as such were maintained without growth factor. Ba/F3 FLT3-WT cells maintained in IL-3 (WT-IL3) express FLT3 but are not dependent on FLT3 signalling, whereas FLT3-WT cells maintained in FLT3 ligand (WT-FL) express FLT3 and are FLT3 dependent.

All lines were routinely confirmed to be free of mycoplasma contamination using the MycoAlert mycoplasma detection kit (Lonza; Basel, Switzerland), as per manufacturer’s instructions. STR analysis for cell line authentication was performed using the Geneprint10 system (Promega; Madison, WI, USA).

**Drugs**

Cell lines and patient samples ex vivo were treated with the following agents, either alone or in combination as indicated in text. DNA-PK inhibitors: NU7441 (SelleckChem; TX, USA), CC115 (Focus Bioscience, St Lucia, QLD, Australia), M3814 (Merck; Darmstadt, Germany); FLT3 inhibitors: Sorafenib (Selleckchem), AC220/Quizartinib (Cayman Chemicals; MI, USA), PKC412/Midostaurin (Novartis; Basel, Switzerland); Cytarabine (SelleckChem), Daunorubicin (SelleckChem). For in vitro use, all inhibitors were dissolved in DMSO; cytarabine and daunorubicin were dissolved in water. For administration to mice, M3814 was suspended in 300mM sodium citrate buffer containing 0.5% methylcellulose and 0.25% Tween 20. Sorafenib was dissolved in 10% Cremophor EL (C5135, Sigma; St Louis, MO, USA) + 10% ethanol.

**Proliferation, apoptosis, and cell cycle analysis**

Cell viability following drug treatments was assessed using the fluorescent viability dye resazurin, as described previously (2). Cells were seeded into 96 well plates, and proliferation following drug treatments was assessed using the fluorescent viability dye Resazurin (excitation 544nm, emission 590nm; 0.6mM Resazurin, 78μM Methylene Blue, 1mM Potassium Hexacyanoferrate (III), 1mM Potassium Hexacyanoferrate (II) Trihydrate (Sigma, St Louis, Missouri, USA), dissolved in sterile PBS). Apoptotic cells were measured using Annexin V-FITC or Annexin V-APC flow cytometry assays (BD Biosciences; NJ, USA), as per manufacturer’s instructions. Cell cycle distribution following drug treatments was assessed using propidium iodide (PI) staining, essentially as described (2). In brief, cells were fixed in 75% ethanol by incubation at 4^o^C. Cells were then washed and incubated in 50ug/mL PI with 1mg/mL RNase for 30min at 37^o^C before flow cytometry analysis.

For combination drug treatments, synergy of dose-response curves was assessed using the method of Chou-Talalay (3), and synergy of individual dose combinations was assessed by the fractional product method of Webb (4).

**Data dependent acquisition (DDA) mass spectrometry (MS)**

Proteins were extracted using the All-in-One kit (Norgen Biotek; Thorold, ON, Canada) followed by acetone precipitation. Protein reduction and alkylation were performed using dithiothreitol and iodoactamide, as previously described (5). In brief, protein samples were suspended in 200uL 6M urea and 2M thiourea. Dithiothreitol was then added to a final concentration of 10mM, and samples were incubated for 20 minutes at room temperature. Cysteine residues were then alkylated by the addition of iodoacetamide to a final concentration of 20mM, followed by incubation for 30 minutes at room temperature. Triethylammonium bicarbonate (TEAB, 50mM, pH7.8) was then added to dilute the urea concentration below 1M, followed by the addition of LysC enzyme 1:30 (w/w) and incubation for 3 hours at room temperature. Trypsin enzyme was then added 1:50 (w/w) and then samples were incubated overnight at room temperature, to enable trypsin digestion. Samples were desalted using Oligo R3 reverse phase material, and 100ug of each sample was individually labelled using 8-plex iTRAQ (Table S1) according to the manufacturer’s instructions (AB Sciex; Foster City, CA, USA). Phosphopeptides were enriched and fractionated using titanium dioxide, sequential elution from IMAC and hydrophobic interaction liquid chromatography (TiSH) (6) as follows. The combined peptide sample was suspended in 80% acetonitrile, 5% TFA, and 1M glycolic acid. TiO_2_ beads were added at 0.6mg per 100ug peptide sample, and the samples were mixed on a shaker at room temperature for 15 minutes. The supernatant was mixed with fresh TiO_2_ beads (0.3mg TiO_2_ beads per 100ug peptide). The two sets of beads with bound phosphopeptides were pooled using 100μL of 80% acetonitrile, 5% TFA, and 1M glycolic acid, followed by washing with 100uL 80% acetonitrile, 1% TFA. The beads were then washed with 10% acetonitrile, 0.1% TFA before being air dried. Phosphopeptides were eluted off the beads with 180μL ammonium hydroxide solution (1% v/v, pH 11.3). The eluted peptides were then speed-vacuumed to dryness before reconstitution in 50μL of 20mM TEAB, pH 6. To remove glycan groups which may be purified using TiO_2_, 2uL glycerol-free PNGase F (New England Biolabs, 500000U/mL, P0705L) and 1μL sialidase A (ProZyme, 5U/mL, GK80040) were added and the sample incubated overnight at 37^o^C. The following day, the sample was diluted with 50% acetonitrile, 0.1% TFA, and the pH adjusted to 1.6-1.8 using 10% TFA. IMAC beads (80μL, Sigma-Aldrich) were washed twice using 200uL 50% acetonitrile, 0.1% TFA, prior to mixing with the peptide solution. The bead/peptide mixture was incubated at room temperature for 30 minutes with gentle shaking to enable binding of phosphorylated peptides. The mixture was then transferred to a 200uL GELoader tip with the end constricted to enable passage of liquid but not beads; liquid was passed through the tip using air pressure applied by a syringe, and was collected into a fresh tube. The IMAC beads within the GELoader tip were washed with 60μL 50% acetonitrile, 0.1% TFA, which was collected with the previous flow through. Mono-phosphorylated peptides were then eluted slowly using 70μL 20% acetonitrile, 1% TFA, and combined with the previous flow through. Multi-phosphorylated peptides were eluted slowly using 80μL ammonium hydroxide solution (1% v/v, pH 11.3). The multi-phosphorylated peptides were then acidified using 10% TFA and desalted with oligo R3 microcolumns. Acetonitrile and TFA was added to the mono-phosphorylated peptide fraction, to final concentrations of 70% and 2%, respectively. Two incubations with TiO_2_ beads were performed as described above. The two bead pellets were combined with 100μL of 50% acetonitrile, 0.1% TFA, and vortexed then centrifuged. The supernatant was aspirated and the beads air-dried for 10 minutes at room temperature. Phosphopeptides were eluted from the beads with ammonium hydroxide solution (1% v/v, pH 11.3), and desalted with oligo R3 microcolumns.

The mono-phosphorylated peptide fraction was reconstituted in 43μL of 90% acetonitrile, 0.1% TFA. The sample was injected onto a capillary HPLC column, using an Agilent 1200 series capillary HPLC system. Elution was performed using a 35 minute gradient of 90% acetonitrile, 0.1% TFA – 60% acetonitrile, 0.1% TFA, and timed fractions were collected into a 96 well plate. A total of 15 fractions were compiled based on the UV detection trace. The fractions were speed vacuumed to dryness, followed by resuspension in 0.1% formic acid and analysis of each fraction by tandem mass spectrometry as described below.

Mass spectrometry was subsequently performed using an Easy-LC nano-HPLC (Proxeon, Odense, Denmark), and an LTQ Orbitrap Velos (Thermo Fisher; Carlsbad, CA, USA). Samples were separated over 120 minutes using a gradient of 0-34% solvent B (solvent A = 0.1% formic acid, solvent B = 90% ACN, 0.1% formic acid) at a flow rate of 250nL/minute. Full MS scans of m/z range 450-1800 were acquired at a resolution of 60,000, with an automatic gain control of 1e6 and maximum injection time 500ms. The top 7 most intense precursor ions were selected for fragmentation using a normalised collision energy of 49. MS/MS scans were acquired using a resolution of 15,000, automatic gain control of 1e4, and maximum injection time of 100ms. Sequest HT and Mascot 2.2.3 search engines were used to search data files against the Uniprot Homo Sapiens proteome (downloaded 16/05/2017, 70,698 sequences) and Swissprot Homo Sapiens proteome databases, respectively. Peptide quantification was normalized to total protein per channel; a localisation probability threshold of 75% was used for determination of peptide modification sites.

Data files were processed using Proteome Discoverer 2.1 (Thermo Fisher) as described (5). Pathway analysis was performed using Ingenuity Pathway Analysis (IPA) software (QIAGEN; Hilden, Germany). For identification of activated kinases, the phosphoproteome of each sample was normalised to its sample median and kinase substrate enrichment analysis (KSEA) was performed using PHOsphosite-X-TRacing Analysis of Causal Kinases (PHOXTRACK) (7). The unweighted data was searched against the Phosphosite Plus database (8) using 1 000 permutations; the minimum number of phosphosites per kinase was 3. Heat maps were generated using MS Excel and Heatmapper (9).

**Parallel reaction monitoring mass spectrometry**

Parallel reaction monitoring (PRM) analysis was performed as previously described (5, 10). Cell pellets were lysed in 0.1M Na_2_CO_3_, pH 11, using a probe sonicator. Reduction, alkylation and digestion was performed as described above. Following digestion, sample pH was adjusted to neutral and deglycosylation enzymes PNGase F and sialidase A were (concentrations as above) and incubated overnight at 37^o^C.

Peptides were desalted using Oasis HLB solid phase extraction (SPE) cartridges and a Visiprep™ SPE Vacuum Manifold (12-port model, Sigma). The SPE cartridges were activated using 100% acetonitrile, then equilibrated using 0.1% TFA. Acidified samples (pH<3) were loaded onto the SPE cartridges and liquid was passed through the solid phase at a drop-wise rate, using vacuum pressure. The cartridges were washed with 0.1% TFA followed by sequential elution of peptides using 60% acetonitrile, 0.1% TFA, and 80% acetonitrile, 0.1% TFA.

Peptides were speed-vacuumed to dryness, then reconstituted in 80% acetonitrile, 5% TFA, and 1M glycolic acid. TiO_2_ beads were added at 0.5mg per 100ug peptide, and the samples incubated with shaking for 15 minutes. The supernatants were added to clean tubes containing 0.3mg TiO_2_ beads per 100ug peptide. The samples were again incubated with shaking and then centrifuged. The supernatants containing non-phosphorylated peptides were removed, and the 2 sets of beads with bound phosphopeptides were pooled using 100μL of 80% acetonitrile, 5% TFA, and 1M glycolic acid. The beads were washed with 100uL 80% acetonitrile, 1% TFA by vortexing for 10 seconds, followed by centrifugation and removal of the supernatant. The beads were then washed in the same manner with 10% acetonitrile, 0.1% TFA. The beads were air-dried for 10 minutes, and then eluted with 180μL ammonium hydroxide solution (1% v/v, pH 11.3). The eluted peptides were then speed-vacuumed to dryness prior to mass spectrometry analysis.

LC Tandem mass spectrometry was performed using a Dionex Ultimate 3000 HPLC, and a Q-Exactive Plus or an Exploris 480 mass spectrometer (Thermo Fisher). Peptides were desalted online with an Acclaim PepMap 100 C18 75uM x 20 mm trap column (Thermo Fisher) prior to separation on a 75uM x 25cm EASY-Spray PepMap C18 column (Q-Exactive Plus, Thermo Fisher) or 75uM x 15cm EASY-Spray PepMap C18 column (Exploris 480, Thermo Fisher) using a 5%-32% acetonitrile gradient (MV4-11 phosphopeptide samples), a 5%-35% acetonitrile gradient (Ba/F3 phosphopeptide samples), or 5%-40% acetonitrile gradient (proteome samples). Using the Q-Exactive Plus, full MS scans of m/z range 400-1300 were acquired at a resolution of 35,000, with an automatic gain control of 1e6 and maximum injection time 50ms. MS/MS scans were acquired using a resolution of 17,500, automatic gain control of 5e5, a normalised collision energy of 30, and maximum injection time of 100ms. Using the Exploris 480, full MS scans of m/z range 370-1500 were acquired at a resolution of 60,000, with an automatic gain control of 1e6 and maximum injection time 50ms. MS/MS scans were acquired using a resolution of 15,000, automatic gain control of 1e6, a normalised collision energy of 30, and maximum injection time of 120ms.

Data files were analysed using PD2.1 (Thermo Fisher) and Skyline (MacCoss Lab; Table S6).

**Human AML xenograft model**

All in vivo experimental procedures were performed following approval from the University of Newcastle Animal Care and Ethics Committee. Female NOD.Cg-Rag1tm1Mom Il2rgtm1Wjl/SzJ (NRG) mice were purchased from the Animal Resources Centre (Perth, Australia), and housed in a specific pathogen-free environment in individually ventilated cages, on a 12 hour light/dark cycle. NRG mice were selected as they are supportive of leukaemia engraftment, but unlike NOD/SCID/IL-2rγnull mice they are wildtype for the mouse DNA-PK gene (*Prkdc*) (11). Mice were given water and standard chow ad libitum. Nine-week old mice were inoculated with MV4-11-luciferase cells (1x10^6^ cells, suspended in 100μL PBS) by injection into the lateral tail vein. Systemic leukaemia burden was assessed by bioluminescence imaging (BLI) using a Xenogen IVIS100 imager, following intraperitoneal injection of luciferin substrate (3mg/mouse; P1043 Promega). Leukaemia burden in the peripheral blood was monitored by flow cytometric analysis of human CD45 (hCD45) proportions. Following erythrocyte lysis using ammonium chloride, white blood cells were stained with anti-human and anti-mouse CD45 antibodies, followed by analysis on a FACS Canto II flow cytometer (BD Biosciences 563879 and Biolegend 103115, respectively).

When the percentage of hCD45 in peripheral blood reached an average of 2% (day 36 post transplantation) the mice were randomly assigned into groups to receive vehicle, 150mg/kg M3814, 5mg/kg sorafenib, or 150mg/kg M3814 + 5mg/kg sorafenib treatment. Treatments were administered by oral gavage once a day (5 days on, 2 days off) for 4 weeks. Mice were monitored daily and were euthanised when they exhibited morbidity symptoms (weight loss, lethargy, inhibited activity).

**Statistical Analysis**

Graphs were produced using Graphpad Prism 7 software (La Jolla, CA, USA). Two sample, unpaired t-tests or one-way ANOVA was used to determine significant differences between groups except where otherwise indicated.

**Data availability**

MS raw data has been deposited to the MassIVE public repository (MSV000084730).

**Supplemental references:**

1. Smith AM, Dun MD, Lee EM, Harrison C, Kahl R, Flanagan H, et al. Activation of the protein phosphatase PP2A in FLT3+ AML cells enhances the cytotoxic activity of FLT3 tyrosine kinase inhibitors. Oncotarget. 2016;7:47465-78.

2. Roberts KG, Smith AM, McDougall F, Carpenter H, Horan M, Neviani P, et al. Essential requirement for PP2A inhibition by the oncogenic receptor c-KIT suggests PP2A reactivation as a strategy to treat c-KIT+ cancers. Cancer Res. 2010;70(13):5438-47.

3. Chou TC. Drug combination studies and their synergy quantification using the Chou-Talalay method. Cancer Res. 2010;70(2):440-6.

4. J. Webb. Effect of more than one inhibitor In: E.R. Hochster. JQ, eds. Enzymes and metabolic inhibitors.: New York: Academic Press; 1963, 487-512.

5. Degryse S, de Bock CE, Demeyer S, Govaerts I, Bornschein S, Verbeke D, et al. Mutant JAK3 phosphoproteomic profiling predicts synergism between JAK3 inhibitors and MEK/BCL2 inhibitors for the treatment of T-cell acute lymphoblastic leukemia. Leukemia. 2017;32:788.

6. Engholm-Keller K, Birck P, Storling J, Pociot F, Mandrup-Poulsen T, Larsen MR. TiSH--a robust and sensitive global phosphoproteomics strategy employing a combination of TiO2, SIMAC, and HILIC. J Proteomics. 2012;75(18):5749-61.

7. Weidner C, Fischer C, Sauer S. PHOXTRACK-a tool for interpreting comprehensive datasets of post-translational modifications of proteins. Bioinformatics (Oxford, England). 2014;30(23):3410-1.

8. Hornbeck PV, Zhang B, Murray B, Kornhauser JM, Latham V, Skrzypek E. PhosphoSitePlus, 2014: mutations, PTMs and recalibrations. Nucleic Acids Res. 2015;43(Database issue):D512-20.

9. Babicki S, Arndt D, Marcu A, Liang Y, Grant JR, Maciejewski A, et al. Heatmapper: web-enabled heat mapping for all. Nucleic Acids Res. 2016;44(W1):W147-53.

10. Dun MD, Chalkley RJ, Faulkner S, Keene S, Avery-Kiejda KA, Scott RJ, et al. Proteotranscriptomic Profiling of 231-BR Breast Cancer Cells: Identification of Potential Biomarkers and Therapeutic Targets for Brain Metastasis. Mol Cell Proteomics. 2015;14(9):2316-30.

11. Barve A, Casson L, Krem M, Wunderlich M, Mulloy JC, Beverly LJ. Comparative utility of NRG and NRGS mice for the study of normal hematopoiesis, leukemogenesis, and therapeutic response. Exp Hematol. 2018;67:18-31.
