# Supplementary material for: Quantitative phosphoproteomics uncovers synergy between DNA-PK and FLT3 inhibitors in acute myeloid leukaemia
Source: Leukemia. 2020 Oct 16;35(6):1782–7. doi: 10.1038/s41375-020-01050-y (PMC8179851; doi:10.1038/s41375-020-01050-y)
Supplement: Supplementary file 2 — Supplementary Figures [file 41375_2020_1050_MOESM2_ESM.pptx]

## Slide 1
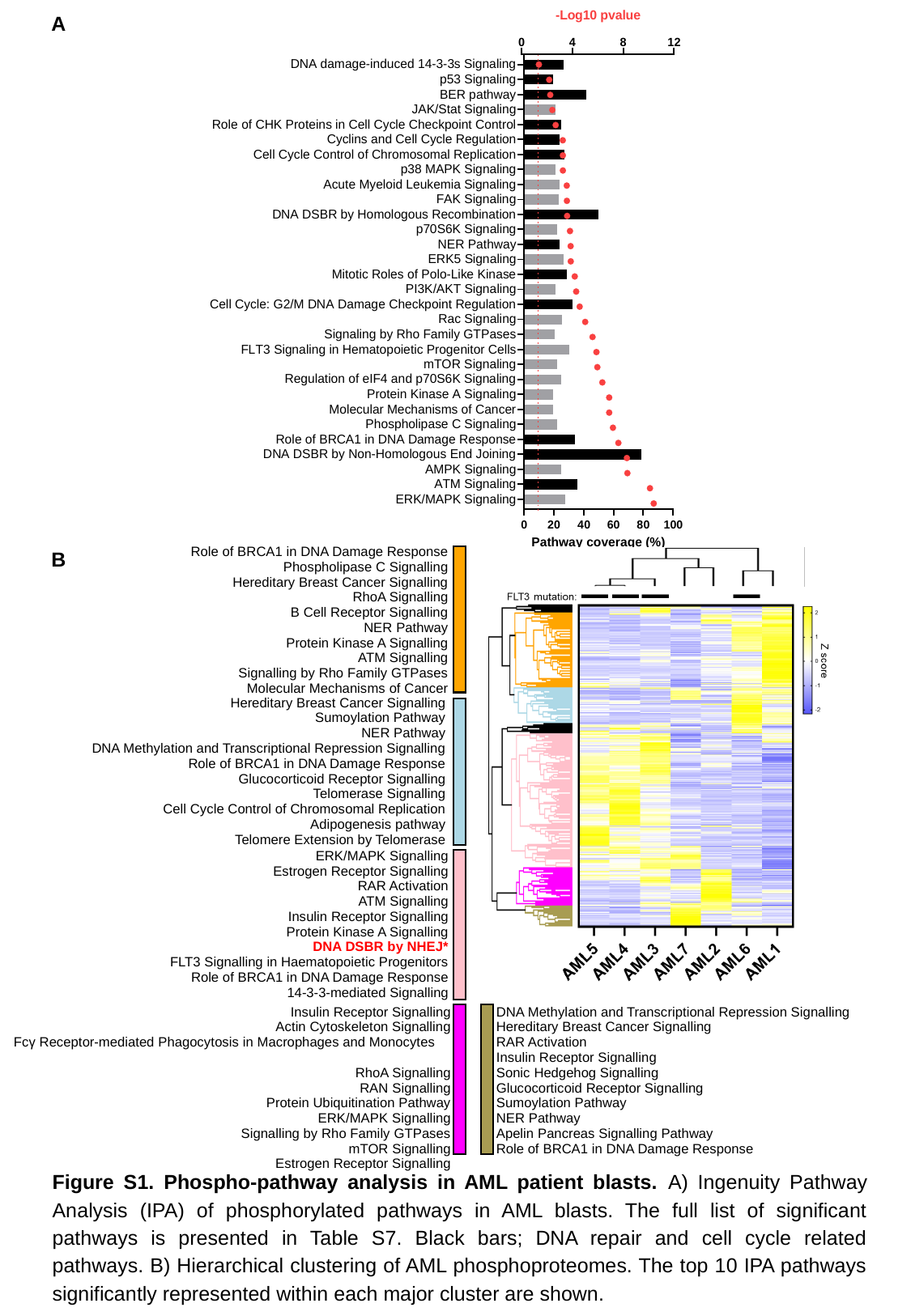

A
Role of BRCA1 in DNA Damage Response
Phospholipase C Signalling
Hereditary Breast Cancer Signalling
RhoA Signalling
B Cell Receptor Signalling
NER Pathway
Protein Kinase A Signalling
ATM Signalling
Signalling by Rho Family GTPases
Molecular Mechanisms of Cancer
Hereditary Breast Cancer Signalling
Sumoylation Pathway
NER Pathway
DNA Methylation and Transcriptional Repression Signalling
Role of BRCA1 in DNA Damage Response
Glucocorticoid Receptor Signalling
Telomerase Signalling
Cell Cycle Control of Chromosomal Replication
Adipogenesis pathway
Telomere Extension by Telomerase
ERK/MAPK Signalling
Estrogen Receptor Signalling
RAR Activation
ATM Signalling
Insulin Receptor Signalling
Protein Kinase A Signalling
DNA DSBR by NHEJ*
FLT3 Signalling in Haematopoietic Progenitors
Role of BRCA1 in DNA Damage Response
14-3-3-mediated Signalling
Insulin Receptor Signalling
Actin Cytoskeleton Signalling
Fcγ Receptor-mediated Phagocytosis in Macrophages and Monocytes
RhoA Signalling
RAN Signalling
Protein Ubiquitination Pathway
ERK/MAPK Signalling
Signalling by Rho Family GTPases
mTOR Signalling
Estrogen Receptor Signalling
DNA Methylation and Transcriptional Repression Signalling
Hereditary Breast Cancer Signalling
RAR Activation
Insulin Receptor Signalling
Sonic Hedgehog Signalling
Glucocorticoid Receptor Signalling
Sumoylation Pathway
NER Pathway
Apelin Pancreas Signalling Pathway
Role of BRCA1 in DNA Damage Response
B
Figure S1. Phospho-pathway analysis in AML patient blasts. A) Ingenuity Pathway Analysis (IPA) of phosphorylated pathways in AML blasts. The full list of significant pathways is presented in Table S7. Black bars; DNA repair and cell cycle related pathways. B) Hierarchical clustering of AML phosphoproteomes. The top 10 IPA pathways significantly represented within each major cluster are shown.

## Slide 2
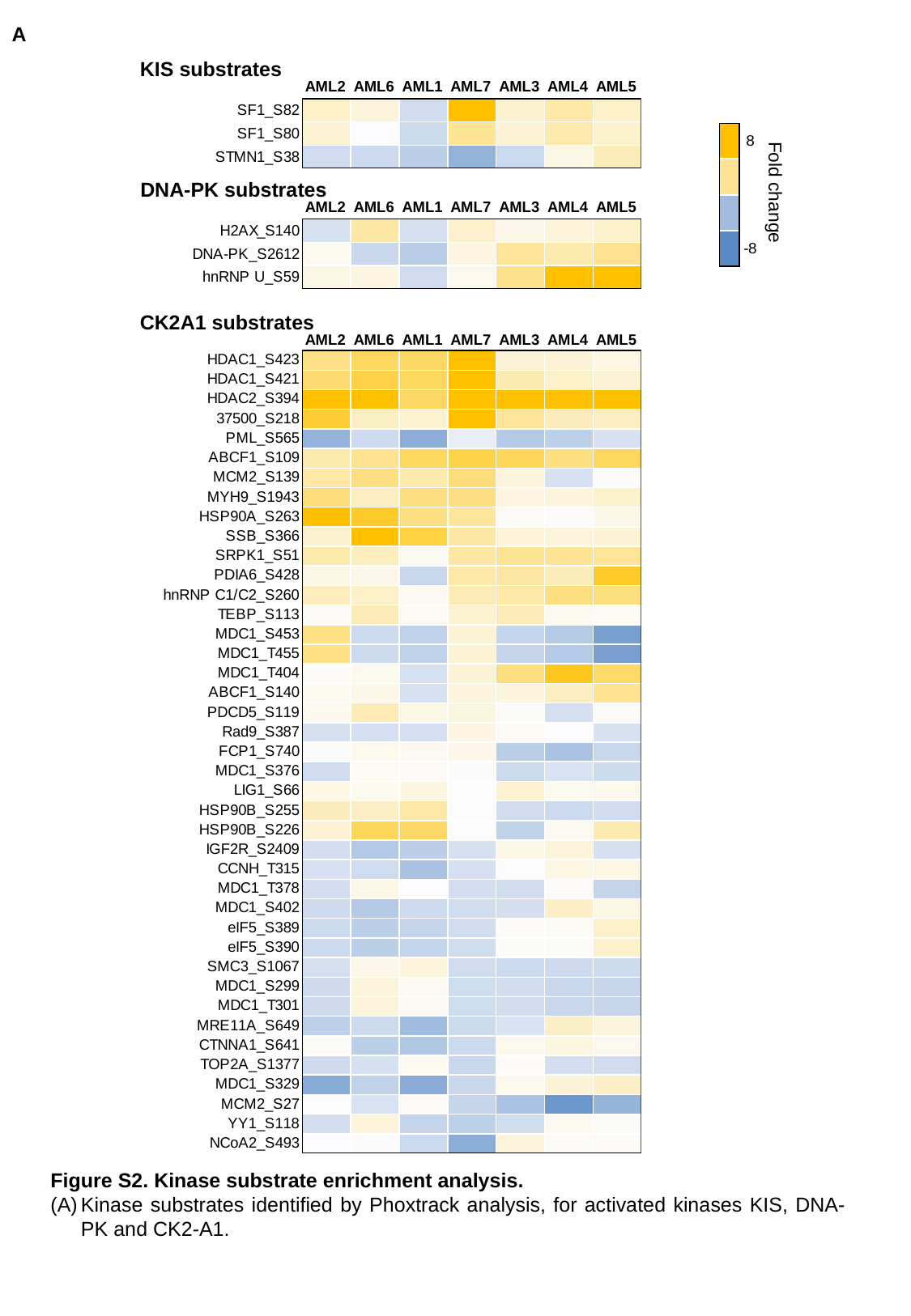

A
KIS substrates
DNA-PK substrates
CK2A1 substrates
Figure S2. Kinase substrate enrichment analysis.
Kinase substrates identified by Phoxtrack analysis, for activated kinases KIS, DNA-PK and CK2-A1.

## Slide 3
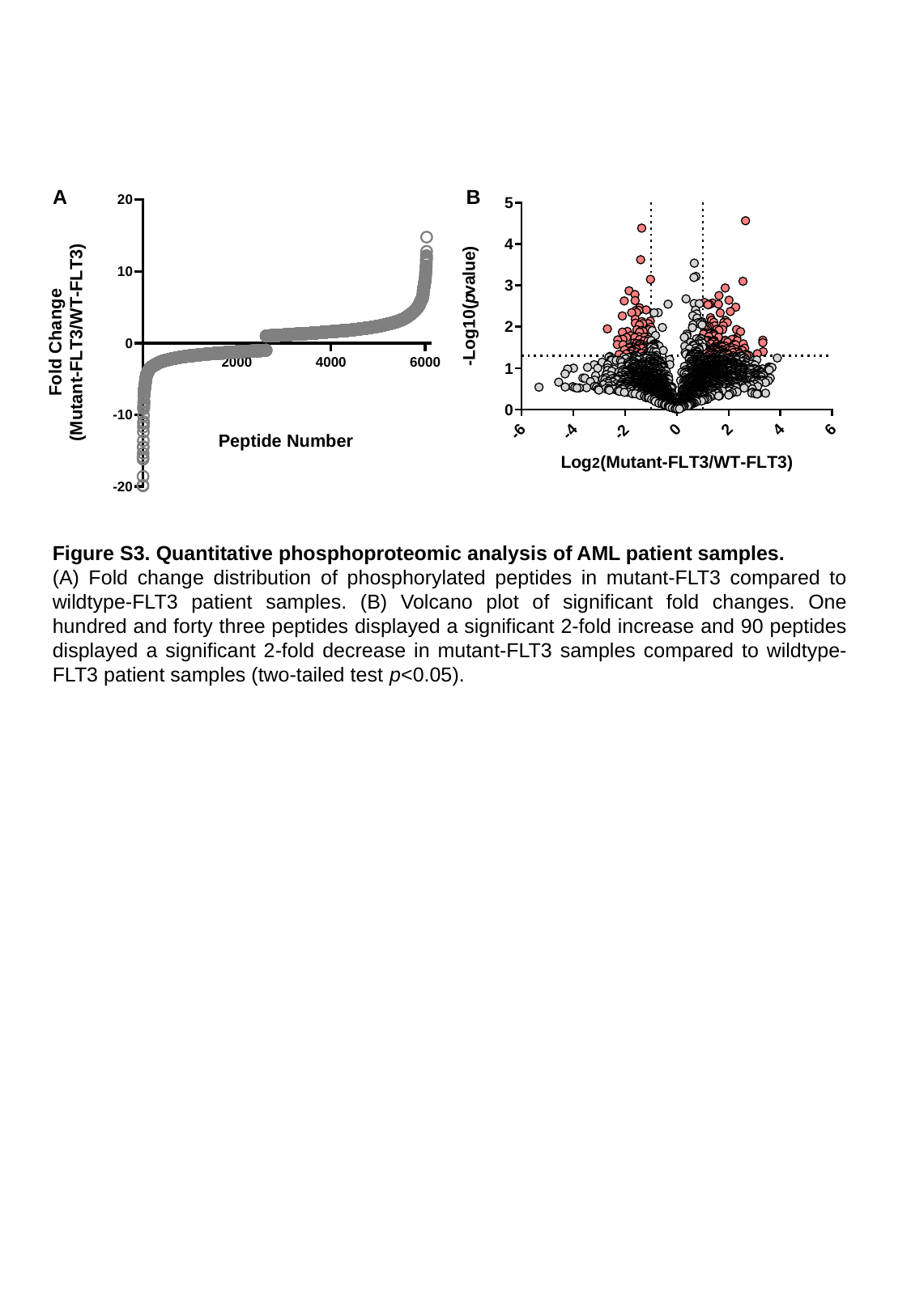

A
B
Figure S3. Quantitative phosphoproteomic analysis of AML patient samples.
(A) Fold change distribution of phosphorylated peptides in mutant-FLT3 compared to wildtype-FLT3 patient samples. (B) Volcano plot of significant fold changes. One hundred and forty three peptides displayed a significant 2-fold increase and 90 peptides displayed a significant 2-fold decrease in mutant-FLT3 samples compared to wildtype-FLT3 patient samples (two-tailed test p<0.05).

## Slide 4
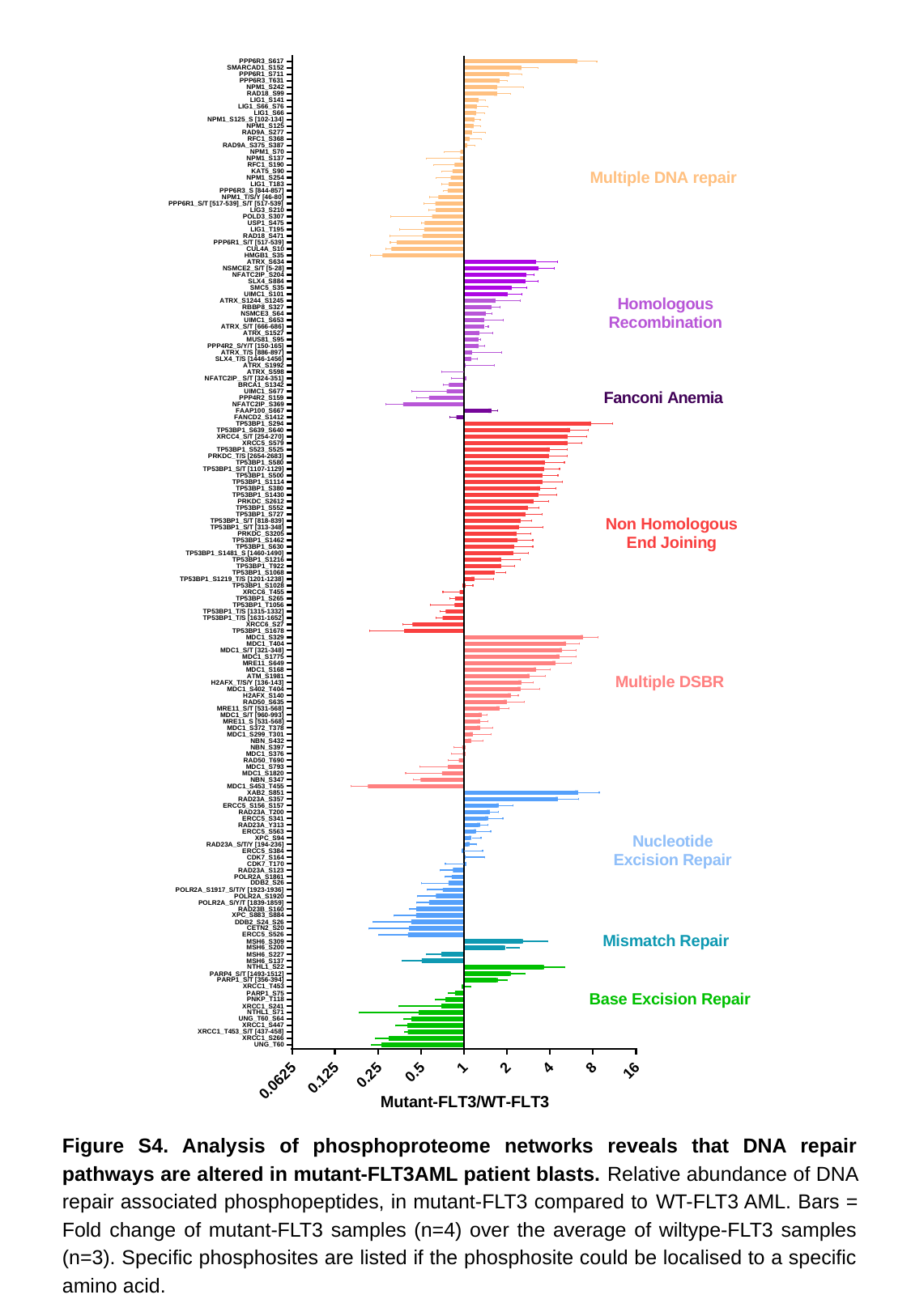

Figure S4. Analysis of phosphoproteome networks reveals that DNA repair pathways are altered in mutant-FLT3AML patient blasts. Relative abundance of DNA repair associated phosphopeptides, in mutant-FLT3 compared to WT-FLT3 AML. Bars = Fold change of mutant-FLT3 samples (n=4) over the average of wiltype-FLT3 samples (n=3). Specific phosphosites are listed if the phosphosite could be localised to a specific amino acid.

## Slide 5
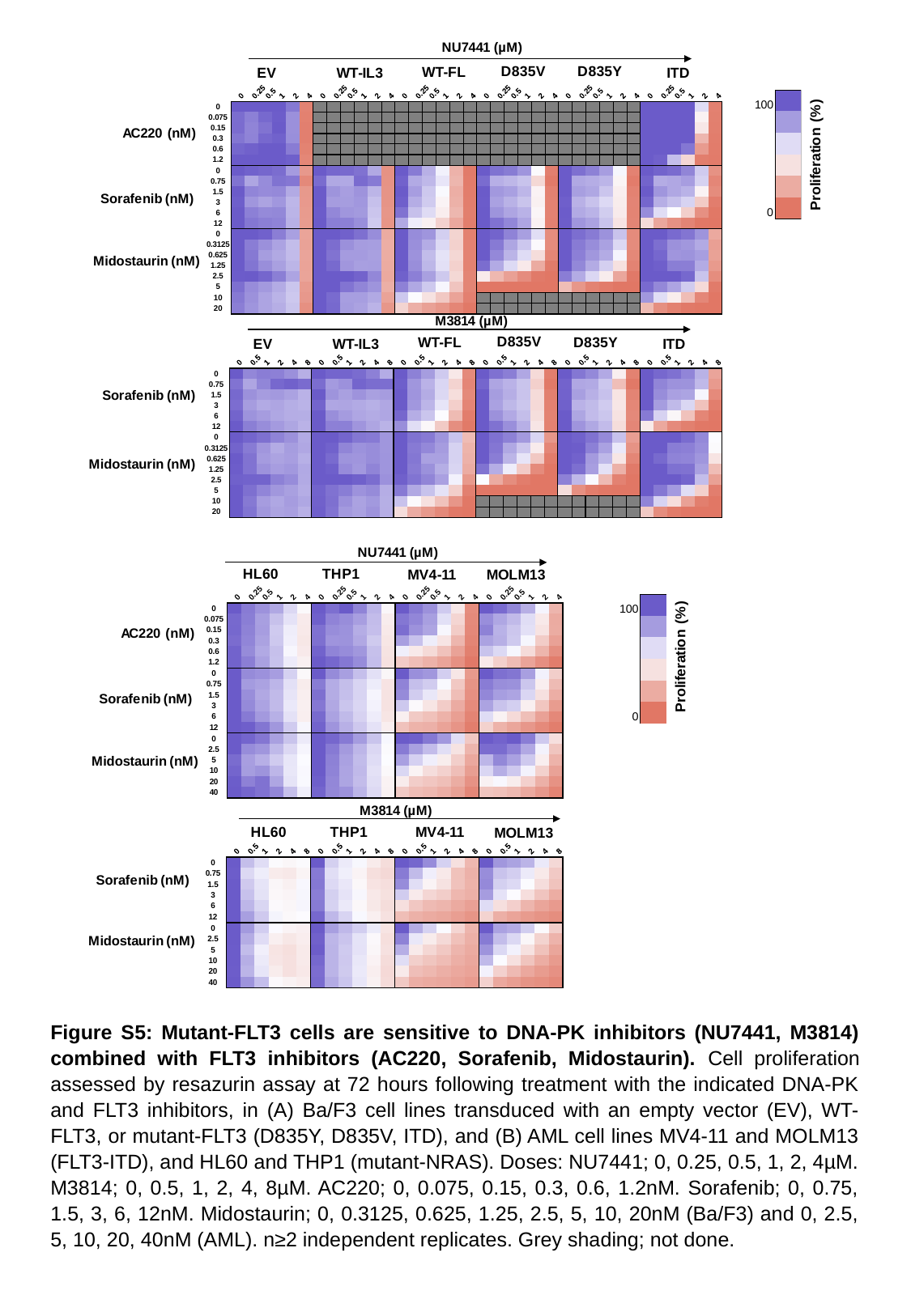

Figure S5: Mutant-FLT3 cells are sensitive to DNA-PK inhibitors (NU7441, M3814) combined with FLT3 inhibitors (AC220, Sorafenib, Midostaurin). Cell proliferation assessed by resazurin assay at 72 hours following treatment with the indicated DNA-PK and FLT3 inhibitors, in (A) Ba/F3 cell lines transduced with an empty vector (EV), WT-FLT3, or mutant-FLT3 (D835Y, D835V, ITD), and (B) AML cell lines MV4-11 and MOLM13 (FLT3-ITD), and HL60 and THP1 (mutant-NRAS). Doses: NU7441; 0, 0.25, 0.5, 1, 2, 4µM. M3814; 0, 0.5, 1, 2, 4, 8µM. AC220; 0, 0.075, 0.15, 0.3, 0.6, 1.2nM. Sorafenib; 0, 0.75, 1.5, 3, 6, 12nM. Midostaurin; 0, 0.3125, 0.625, 1.25, 2.5, 5, 10, 20nM (Ba/F3) and 0, 2.5, 5, 10, 20, 40nM (AML). n≥2 independent replicates. Grey shading; not done.

## Slide 6
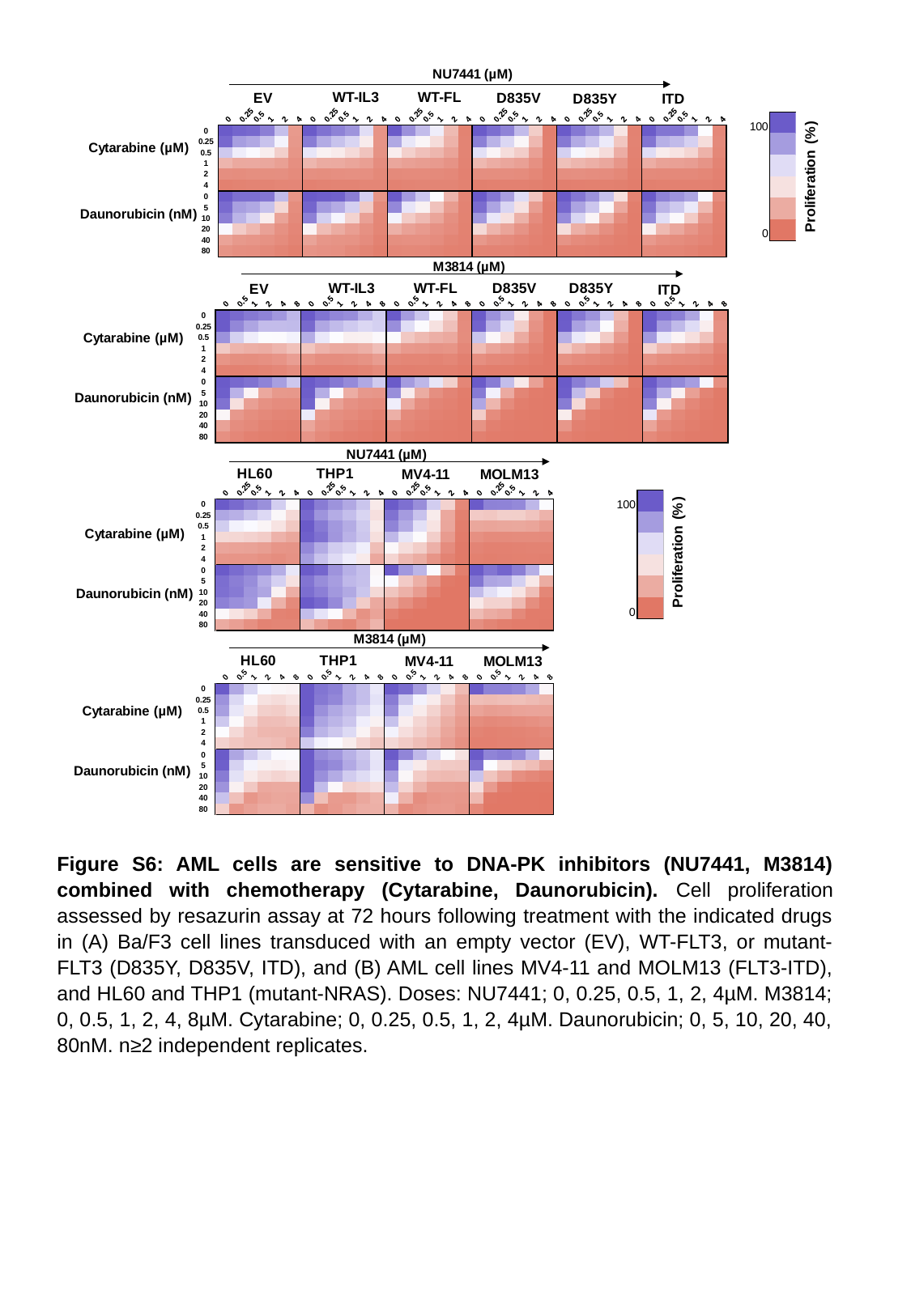

Figure S6: AML cells are sensitive to DNA-PK inhibitors (NU7441, M3814) combined with chemotherapy (Cytarabine, Daunorubicin). Cell proliferation assessed by resazurin assay at 72 hours following treatment with the indicated drugs in (A) Ba/F3 cell lines transduced with an empty vector (EV), WT-FLT3, or mutant-FLT3 (D835Y, D835V, ITD), and (B) AML cell lines MV4-11 and MOLM13 (FLT3-ITD), and HL60 and THP1 (mutant-NRAS). Doses: NU7441; 0, 0.25, 0.5, 1, 2, 4µM. M3814; 0, 0.5, 1, 2, 4, 8µM. Cytarabine; 0, 0.25, 0.5, 1, 2, 4µM. Daunorubicin; 0, 5, 10, 20, 40, 80nM. n≥2 independent replicates.

## Slide 7
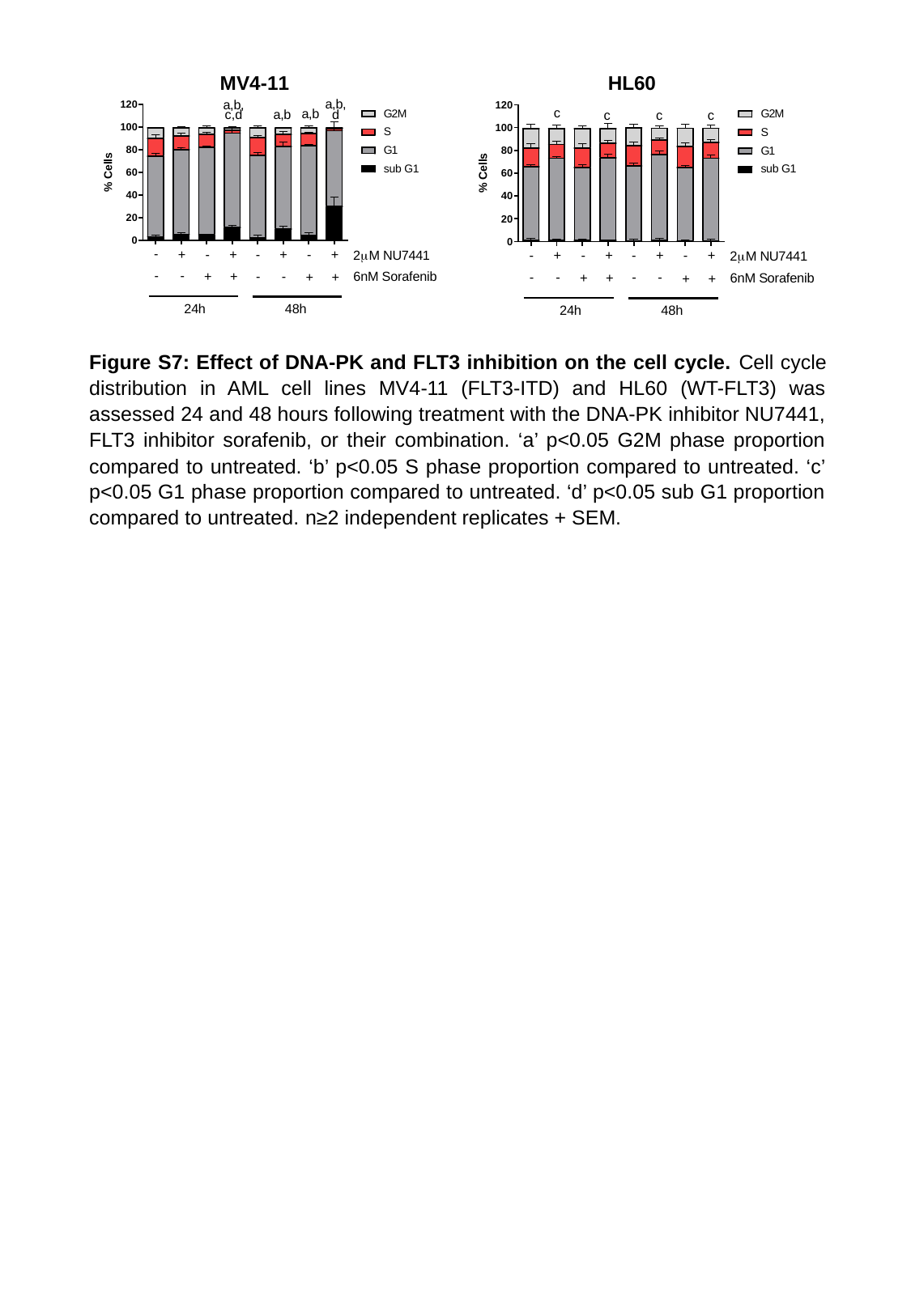

Figure S7: Effect of DNA-PK and FLT3 inhibition on the cell cycle. Cell cycle distribution in AML cell lines MV4-11 (FLT3-ITD) and HL60 (WT-FLT3) was assessed 24 and 48 hours following treatment with the DNA-PK inhibitor NU7441, FLT3 inhibitor sorafenib, or their combination. ‘a’ p<0.05 G2M phase proportion compared to untreated. ‘b’ p<0.05 S phase proportion compared to untreated. ‘c’ p<0.05 G1 phase proportion compared to untreated. ‘d’ p<0.05 sub G1 proportion compared to untreated. n≥2 independent replicates + SEM.

## Slide 8
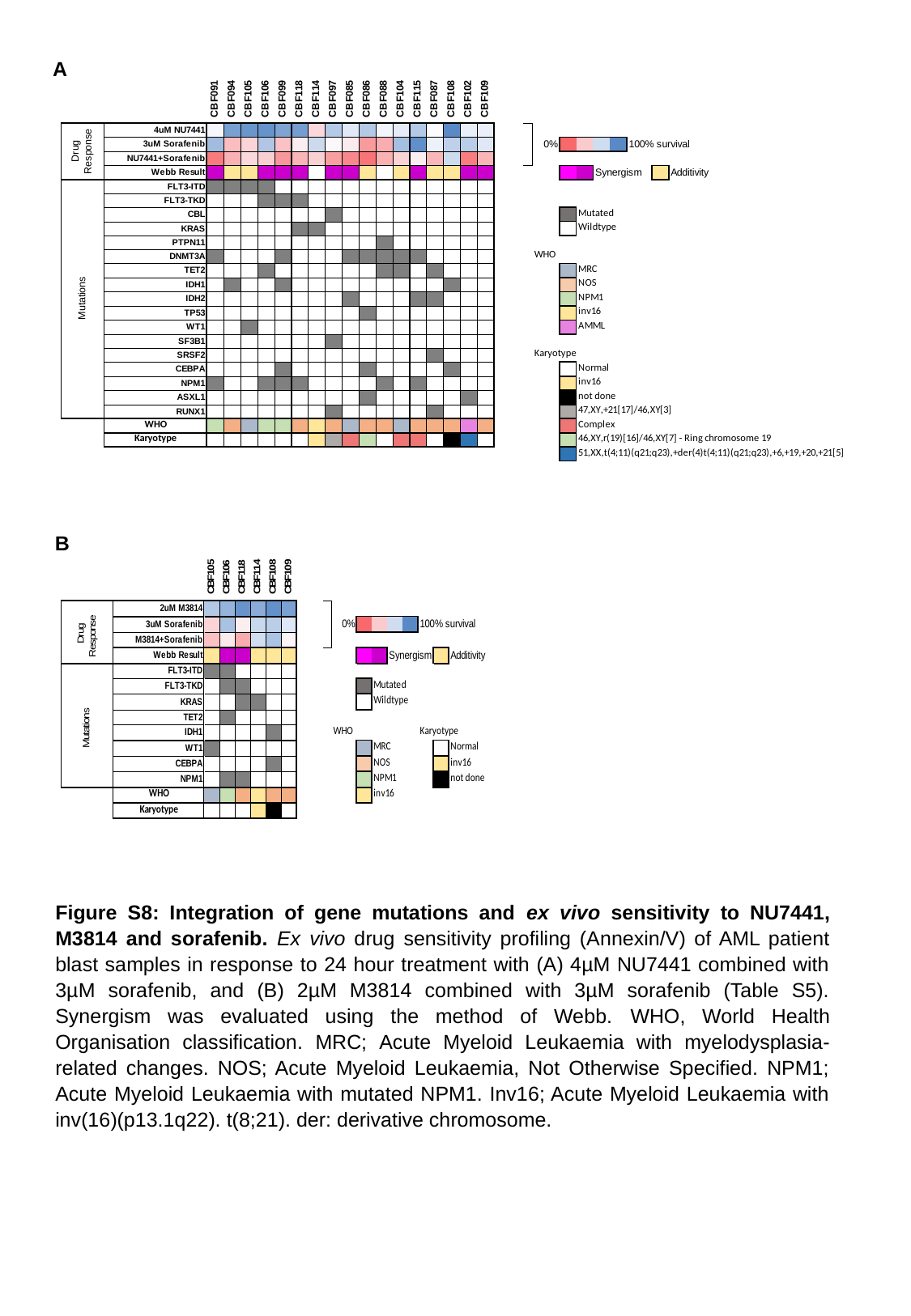

A
B
Figure S8: Integration of gene mutations and ex vivo sensitivity to NU7441, M3814 and sorafenib. Ex vivo drug sensitivity profiling (Annexin/V) of AML patient blast samples in response to 24 hour treatment with (A) 4µM NU7441 combined with 3µM sorafenib, and (B) 2µM M3814 combined with 3µM sorafenib (Table S5). Synergism was evaluated using the method of Webb. WHO, World Health Organisation classification. MRC; Acute Myeloid Leukaemia with myelodysplasia-related changes. NOS; Acute Myeloid Leukaemia, Not Otherwise Specified. NPM1; Acute Myeloid Leukaemia with mutated NPM1. Inv16; Acute Myeloid Leukaemia with inv(16)(p13.1q22). t(8;21). der: derivative chromosome.

## Slide 9
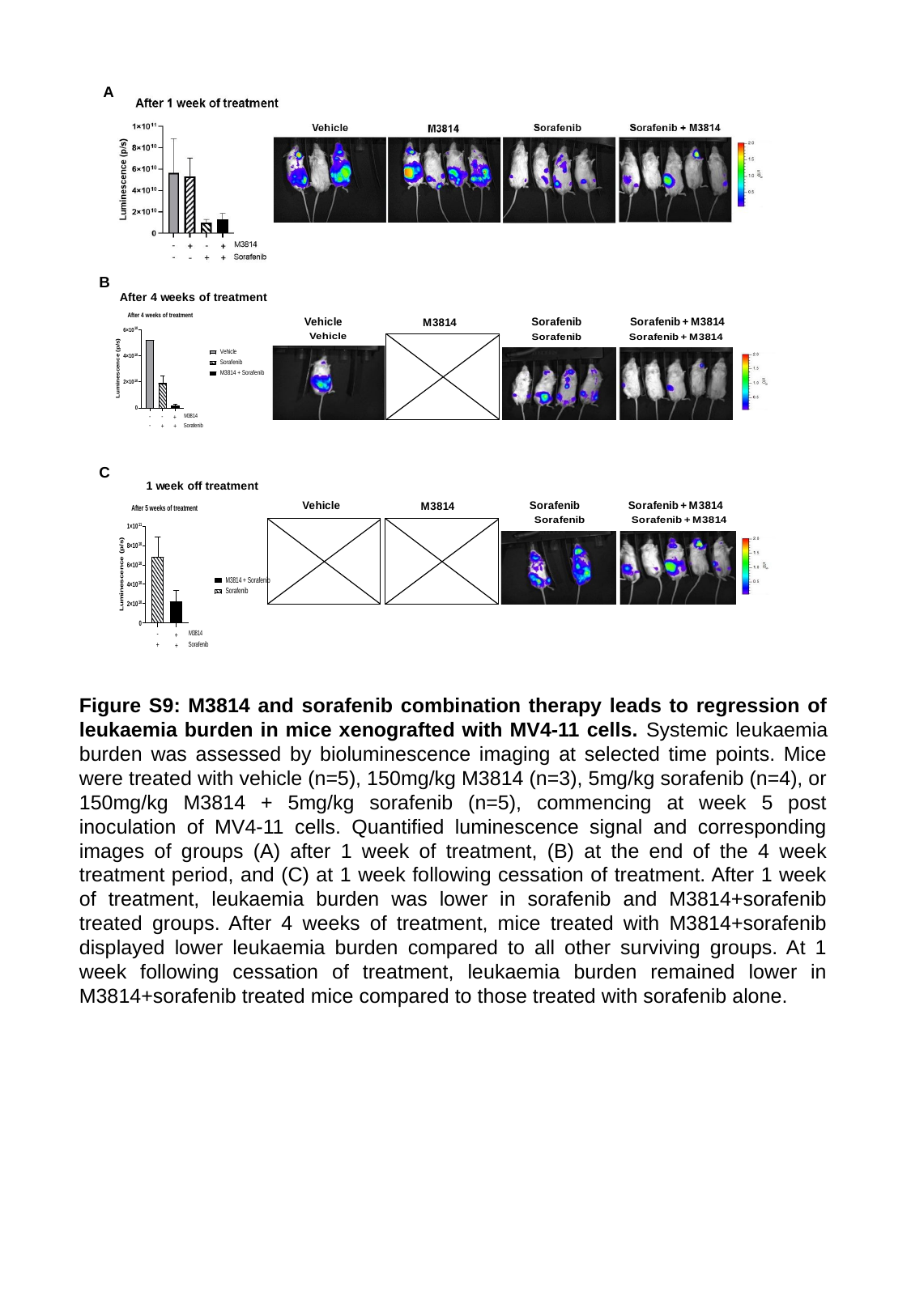

Figure S9: M3814 and sorafenib combination therapy leads to regression of leukaemia burden in mice xenografted with MV4-11 cells. Systemic leukaemia burden was assessed by bioluminescence imaging at selected time points. Mice were treated with vehicle (n=5), 150mg/kg M3814 (n=3), 5mg/kg sorafenib (n=4), or 150mg/kg M3814 + 5mg/kg sorafenib (n=5), commencing at week 5 post inoculation of MV4-11 cells. Quantified luminescence signal and corresponding images of groups (A) after 1 week of treatment, (B) at the end of the 4 week treatment period, and (C) at 1 week following cessation of treatment. After 1 week of treatment, leukaemia burden was lower in sorafenib and M3814+sorafenib treated groups. After 4 weeks of treatment, mice treated with M3814+sorafenib displayed lower leukaemia burden compared to all other surviving groups. At 1 week following cessation of treatment, leukaemia burden remained lower in M3814+sorafenib treated mice compared to those treated with sorafenib alone.
